# Decoding HIV‐1 Next Move Through Matrix Protein p17 Quasi‐Species

**DOI:** 10.1002/mbo3.70280

**Published:** 2026-04-03

**Authors:** Serena Messali, Anna Bertelli, Marta Giovanetti, Leonardo Sclavi, Massimo Ciccozzi, Mark Slevin, Arnaldo Caruso, Francesca Caccuri

**Affiliations:** ^1^ Section of Microbiology, Department of Molecular and Translational Medicine University of Brescia Brescia Italy; ^2^ Division of Microbiology ASST‐Spedali Civili Brescia Italy; ^3^ Sciences and Technologies for Sustainable Development and One Health University of Campus Bio‐Medico Rome Italy; ^4^ Instituto René Rachou Fundação Oswaldo Cruz Belo Horizonte Minas Gerais Brazil; ^5^ Unit of Medical Statistics and Molecular Epidemiology University of Campus Bio‐Medico Rome Italy; ^6^ Center for Advanced Medical and Pharmaceutical Research (CCAMF) George Emil Palade University of Medicine, Pharmacy, Science and Technology Târgu Mures Romania

**Keywords:** AIDS‐related lymphomas, clonogenic p17 variants, HIV‐1 matrix protein p17, HIV‐1 mutants, viral quasi‐species, virus evolution

## Abstract

Since the introduction of combined antiretroviral therapy, acquired immune deficiency syndrome (AIDS)‐related lymphomas account for a growing proportion of deaths among people living with human immunodeficiency virus (PLWHIV). In addition to the immune deficiency caused by AIDS and other cofactors, it has been shown that circulating HIV‐1 proteins play a critical role in lymphoma development. The HIV‐1 matrix protein p17 (refp17) is released from infected cells and accumulates in lymph nodes of PLWHIV, even during effective pharmacological control of viral replication. Circulating refp17 deregulates the biological activity of different immune cells. Moreover, p17 variants (vp17s) characterized by peculiar amino acid insertions occurring in the C‐terminal region of the protein, differently from the refp17, also induce B‐cell growth and clonogenicity. Notably, vp17s were found at a significantly higher prevalence in PLWHIV with than without lymphoma. HIV‐1 mutants expressing clonogenic vp17s are actively spreading, and their prevalence is globally increasing worldwide. RNA viruses exist as a population of quasi‐species, transmitted from one host to another, which ultimately leads to viral evolution by generating new master sequences. Here, we developed a next‐generation sequence approach to evaluate the frequency of vp17 quasi‐species in PLWHIV upon time and demonstrated that the incidence of vp17s also increases at quasi‐species levels. Additionally, we established a regression model capable of predicting the insertions with higher probability to be fixed, further highlighting the evolutionary relevance of the C‐terminal region in the adaptation of p17 to the human host.

## Graphical abstract

HIV‐1 mutants expressing clonogenic HIV‐1 matrix protein p17 variants (vp17s) are spreading, increasing their prevalence worldwide. In RNA viruses, quasi‐species are transmitted among hosts, leading to the generation of new master sequences. We developed a next‐generation sequence approach to evaluate the frequency of vp17 quasi‐species in people living with human immunodeficiency virus, demonstrating their increased incidence.

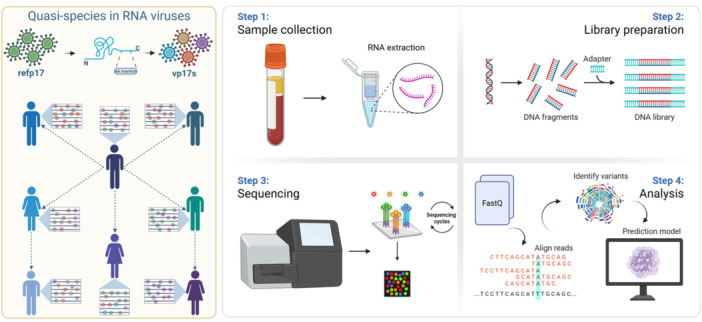

## 1 Introduction

The advent of combined antiretroviral therapy (cART) led to a prolonged overall life expectancy of people living with human immunodeficiency virus (PLWHIV) and to a significantly decline in progression to acquired immune deficiency syndrome (AIDS). As a consequence, the incidence of many cancers among PLWHIV decreased, but their risk to develop Hodgkin and non‐Hodgkin lymphoma still stays significantly higher than the general population (Odeny et al. 2024). Up to date, AIDS‐related lymphomas (ARLs) account for a growing proportion of deaths among PLWHIV (Liang et al. 2025; Noy 2019), and remain a major clinical challenge due to their high incidence, extra‐nodal involvement, and resistance to canonical treatments (Wang et al. 1995; Sirsath et al. 2013). The median overall survival for ARLs in PLWHIV ranges from 6 to 24 months, resulting remarkably shorter as compared with HIV‐1‐seronegative lymphoma patients treated with equal therapy (Cingolani et al. 2017; Chow et al. 2001).

Besides the immune deficiency caused by AIDS, B‐cell activation and coinfection with virus‐associated tumors, such as Epstein–Barr virus, Hepatitis B virus, Hepatitis C virus, and Human Papillomavirus (Lurain et al. 2022; Chao et al. 2012), a huge amount of data demonstrated the critical role played by circulating HIV‐1 proteins in lymphoma pathogenesis (Caccuri et al. 2019; Curreli et al. 2013). Indeed, it is well established that the production and release of HIV‐1 Gag proteins is sustained by cells harboring latent viruses or defective proviruses, also in the absence of active virus replication (Imamichi et al. 2020; Pollack et al. 2017).

The HIV‐1 matrix protein p17 (p17) is a Gag‐encoded protein exerting complex functions in the virus life cycle (Caccuri, Giagulli, et al. 2022; Fiorentini et al. 2006). It is released from infected cells (Caccuri et al. 2016) and accumulates in lymph nodes of PLWHIV, even during pharmacological control of viral replication (Caccuri, Messali, et al. 2022; Caccuri et al. 2014; Dolcetti et al. 2015). Circulating p17, at nanomolar concentrations, has been detected in sera of both viremic and aviremic PLWHIV (Zani, Messali, Uggeri, et al. 2024) and found to deregulate the biological activity of different cells involved in HIV‐1 pathogenesis (Fiorentini et al. 2010). Differently from the wild‐type p17 (refp17), p17 variants (vp17s) characterized by peculiar amino acid (aa) insertions—mainly occurring at the C‐terminal region at positions 114–115, 117–118, and/or at positions 125–126—were found to be able of triggering B‐cell growth and clonogenicity (Dolcetti et al. 2015; Caccuri, Messali, et al. 2022). This gain‐of‐function is due to a protein misfolding (Giagulli et al. 2011; Dolcetti et al. 2015), caused by aa insertions, which leads to the exposure of a functional epitope located in vp17s N‐terminal portion (He et al. 2019; D'Ursi et al. 2024). The interaction between the functional epitope and the protease‐activated receptor 1 (PAR‐1), followed by epithelial growth factor receptor transactivation and activation of the phosphoinositide 3‐kinases/protein kinase B (PI3K/Akt) pathway, has been found to be responsible for B‐cell growth‐promoting activity (Caccuri et al. 2014, Giagulli et al. 2021).

It has been recently shown that the generation of clonogenic vp17s is due to an imperfect homologous recombination events occurring in the *Gag* gene, at the end of matrix protein and in proximity of hot spots of mutations (Zani, Messali, Bugatti et al. 2024). It is worth noting that vp17s were found at a significantly higher prevalence in PLWHIV with than without lymphoma (Dolcetti et al. 2015), and more interestingly, HIV‐1 mutants expressing clonogenic vp17s are actively spreading and their prevalence is globally increasing over time (Caccuri, Messali, et al. 2022).

In a single host, RNA viruses exist as quasi‐species, a dynamic population of genetically related nonidentical viruses continuously reshaping in reaction to a changing environment, which allows persistence and adaptation of viruses to host and therapy (Westfall et al. 2024). This ultimately leads to viral evolution by generating new master sequences (MS) (Domingo and Perales 2019).

Quasi‐species transmission is regulated by the bottleneck event, which controls the number of quasi‐species transmitted from one host to another to establish an infection and, based on its size, it significantly regulates the genetic diversity of the virus (McCrone and Lauring 2018; Zwart and Elena 2015). A narrow transmission bottleneck restricts viral diversity, while a wide one allows an increased frequency of transmitted quasi‐species (Joseph et al. 2015; Keele et al. 2008).

Although most proviruses in the HIV‐1 reservoir remain latent during cART, a small fraction is transcriptionally active and sustains low levels of viremia (LLV). LLV is present in about 30% of PLWHIV receiving cART, and it has been reported to contribute to rebound viremia following treatment interruption (Aamer et al. 2020; Santoro et al. 2014; World Health Organization 2016; Zhang et al. 2020). A detailed analysis of LLV showed viral evolution by accumulation of aa mutations and the constitution of larger reservoirs as compared with subjects without LLV (Tobin et al. 2005; Lorenzo‐Redondo et al. 2016; Bachmann et al. 2019). Therefore, a small fraction of transcriptionally active proviruses in the HIV‐1 reservoir is likely to contribute to the appearance of new circulating HIV‐1 mutants. Viral MSs in LLV were reported to be similar to those found in the early stages of HIV‐1 infection (Nettles et al. 2005). However, while reservoir diversity of viral MS did not differ significantly between the pre‐ and post‐LLV, the composition of viral quasi‐species was found markedly changed (Sun et al. 2024), suggesting that quasi‐species may ultimately be the source of new viral mutants displaying a better fitness to the human host. Altogether, these evidences highlight the importance of a wide genomic surveillance to follow evolution and spreading of HIV‐1 mutants worldwide. Indeed, incidence evaluation of HIV‐1 mutants expressing clonogenic vp17s can be informative for understanding the potential risk of lymphoma development in PLWHIV, allowing the implementation of prevention strategies aimed at reducing cancer incidence rates in this population.

In this study, we evaluated the frequency of vp17 quasi‐species in PLWHIV over time to understand whether an increase in their incidence might have mediated the generation of new MS. To this end, we developed a next‐generation sequence (NGS) approach aimed to evaluate the incidence of clonogenic vp17s at quasi‐species levels and compared samples from an old cohort of PLWHIV with those obtained in the most recent decade. We demonstrated that the incidence of vp17s also increases at quasi‐species levels, possibly leading to the generation of new mutant MS over time. Finally, with the aim of identifying the insertions with the greatest probability of fixation, we designed a regression model capable of recognizing evolutionary patterns associated with fixed insertions, which highlighted the evolutionary relevance of the C‐terminal region in the adaptation of p17 to the human host.

## 2 Materials and Methods

### 2.1 Patients

A total of 118 plasma samples from HIV‐1‐infected patients treated at the Centro di Riferimento Oncologico, NCI, Aviano, Italy, or undergoing routine monitoring of viremia at the Brescia Civic Hospital (BS), Italy, were collected from 1997 to 2020 and divided into two distinct cohorts. The < 2010 included 65 plasma samples collected from 1997 to 2010, while the > 2010 cohort included 53 residual plasma samples obtained from 2017 to 2020. All patients were infected with HIV‐1 subtype B as determined by the sequence analysis of the p17 region, according to the Los Alamos genotyping algorithm. The study was conducted in accordance with the Declaration of Helsinki and national standards, and was approved by the Brescia Ethics Committee (NP 3163). Before starting the analyses, the samples were completely anonymized.

### 2.2 RNA Extraction, Reverse Transcription, and cDNA Amplification

RNA was extracted from 200 μL of plasma, using the QIAamp DSP Virus kit (QIAGEN, Heiden, Germany) according to the manufacturer's instructions, and it was eluted in 30 μL Buffer AVE. The p17 viral gene was reverse transcribed, and polymerase chain reaction (PCR) amplified employing the SuperScript III One‐Step RT‐PCR system with PlatinumTaq DNA polymerase (Thermo Fisher Scientific, Carlsbad, CA, USA) in a 50‐μL reaction containing 25 μL of reaction mix, 9 μL of MgSO_4_, 2 μL of SuperScript III RT/PlatinumTaq Mix, 0.2 μM of sense and antisense primers, and 12 μL of extracted RNA. The amplification conditions were as follows: 50°C for 30 min (for reverse transcription) and 94°C for 2 min for Taq DNA polymerase activation, followed by 40 cycles (94°C 15 s, 55°C 30 s, and 68°C 2 min) and a final cycle at 68°C 7 min. The PCR primers used in the reaction were UGF1, 5′‐GTGCCCGTCTGTTGTGTG and p24R1, 5′‐CATTTGCATGGCTGCTTGATG. Afterwards, to evaluate the correct amplification, PCR products were checked on a 1.0% agarose gel, purified using the QIAquick PCR Purification Kit (QIAGEN), and stored at −20°C until use.

### 2.3 NGS and Quasi‐Species Analysis

An amplicon‐based approach was chosen to amplify a 153‐nucleotide‐long amplicon that covered the COOH‐terminal region of the p17 gene. For library preparation, 5 μL of PCR products was amplified to incorporate Illumina adaptor sequences using the AmpliTaq Gold DNA Polymerase (Thermo Fisher Scientific, Carlsbad, CA, USA) in a total volume of 50 μL containing each primer with the overhang adapter at 0.15 μM (p17Mini_fw 5′‐TCG TCG GCA GCG TCA GAT GTG TAT AAG AGA CAG GAC ACC AAG GAA GCC TTA GAT AAG and p24Mini_rv 5′‐GTC TCG TGG GCT CGG AGA TGT GTA TAA GAG ACA GCC TGA TGT ACC ATT TGC CCC TG). PCR conditions were performed as follows: 95°C for 10 s, 30 cycles at 94°C for 15 s, 59°C for 30 s, and 72°C for 2 min, followed by a final extension at 72°C for 7 min. After checking on a 1.5% agarose gel, PCR products were purified using Agencourt Ampure XP beads (Beckman Coulter) at the ratio of 1.8. The beads were washed twice with 80% ethanol, and the PCR products were eluted in 30 μL of 10 mM Tris, pH 8.5. Then, 7 μL of the purified first‐round PCR products were used for the second‐round necessary to add Illumina sequencing adapters and dual‐index barcodes, performed with the AmpliTaq Gold DNA Polymerase. After eight cycles of PCR amplification with indexed primers (IDT Technologies, Leuven, Belgium), libraries were checked on a 1.5% agarose gel and cleaned up using AMPure XP beads. Purified libraries were quantified with the Qubit Fluorometer (Qubit DNA HS Assay Kit, Thermo Fisher Scientific), pooled, and loaded in a V2 300‐cycle sequencing cartridge to perform sequencing on the MiSeq platform (Illumina). Raw data were checked for quality using FastQC (https://www.bioinformatics.babraham.ac.uk/projects/fastqc/). The paired‐end reads were trimmed with Trimmomatic version 0.39 for quality (*Q* score > 30) and length (> 100 bp), and the derived sequences were analyzed and edited with Geneious software (version 11.1.5) (Biomatters Ltd., New Zealand). A consensus sequence was reconstructed by mapping the reads to the pNL4.3 reference sequence (Accession number AF324493) using Bowtie2 in high sensitivity‐local mode.

To evaluate the occurrence of a single aa or multiple aa insertions at the p17 quasi‐species level, all the mapped sequencing reads were translated and realigned. The presence of the aa insertions was manually checked, and the percentage of mapped reads carrying the inserted residues was calculated. Of note, a quasi‐species was considered significant when the aa insertion was represented in at least 5% of the mapped reads.

### 2.4 Predictive Model for vp17 Evolution

Data derived from sequences containing insertions—including positional information, mutation types, and relative frequencies—were used to train a predictive model based on logistic regression (with the option to apply a Random Forest classifier). The model was trained to learn the evolutionary patterns that characterize already fixed mutations and subsequently applied to refp17 MS sequences to estimate, for each quasi‐species, the probability of future fixation. Mutations were then combined in pairs or triplets and ranked according to their combined fixation risk, calculated as $p_{\mathrm{comb}}=1-\prod_{i}(1-p_{i})$ assuming first‐order independence among events.

### 2.5 Statistical Analysis

Data were analyzed for statistical significance using the Chi‐square test. Differences were considered significant at *p* < 0.05. Statistical tests were performed using GraphPad Prism 8 software (GraphPad).

## 3 Results

### 3.1 Cohort Characteristics

**Figure 1:**
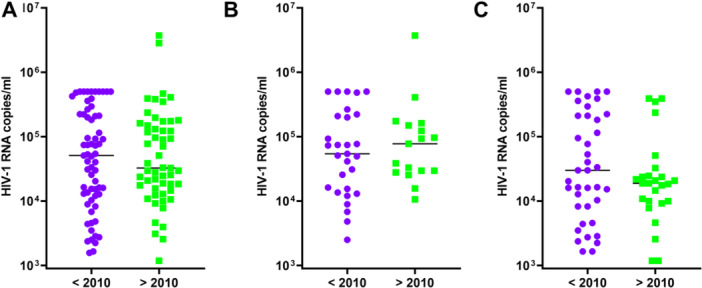
HIV‐1 viral load levels in enrolled patients. The viral load in the < 2010 cohort (purple dots) and in the > 2010 one (green squares) is represented in relation to the administration of cART therapy. HIV‐1 viral loads levels, expressed as HIV‐1 RNA copies/ml, (A) in the totality of the enrolled PLWHIV, (B) in the naïve PLWHIV, and (C) in the experienced PLWHIV patients. cART, combined antiretroviral therapy; PLWHIV, people living with human immunodeficiency virus.

Our previous study showed, for the first time, the existence of vp17s in a cohort of patients collected between 1997 and 2010 (Dolcetti et al. 2015; Caccuri, Messali, et al. 2022). To evaluate the incidence of vp17s in a more recent group of PLWHIV, we compared 65 samples of the previous cohort with 53 samples collected from 2010 to 2020. The majority (72%) were male, and the median (interquartile range) age was 46 (37.75–53.50) years. All patients were viremic and included 46 patients never subjected to cART treatment (naïve) and 53 patients which underwent cART treatment failure (experienced), only for 19 patients we did not have information about cART treatment. On the whole, viremia of the 65 patients belonging to the < 2010 cohort was similar to that observed for the 53 participants belonging to the > 2010 one (Figure 1A). Analysis of the viral load among naïve (Figure 1B) or experienced (Figure 1C) patients of the two cohorts did not display statistically significant differences.

### 3.2 HIV‐1 Virions Expressing vp17 Quasi‐Species Are Increasing over Time

**Figure 2:**
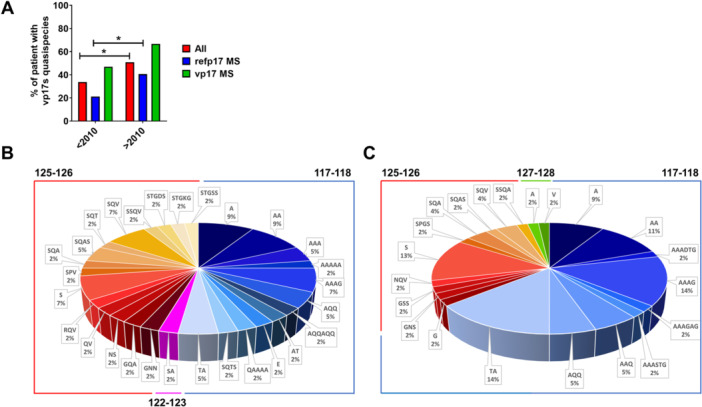
Occurrence of vp17 quasi‐species over time. (A) Percentage of PLWHIV expressing vp17 quasi‐species within the < 2010 cohort and the > 2010 one. Red bars indicate the totality of the enrolled PLWHIV endowing vp17s at quasi‐species level, blue bars show the percentage of PLWHIV endowing refp17 MS and harboring quasi‐species, the green bars represent the percentage of PLWHIV showing vp17s in the MS and carrying quasi‐species. Statistical significance was calculated with the Chi‐square test. (B, C) Pie charts representing the frequency of aa insertion in the totality of PLWHIV with vp17 quasi‐species within (B) the < 2010 cohort, and (C) the > 2010 one. Each insertion position is identified by a color code: position 117–118 in blue, 122–123 in pink, 125–126 in red, and 127–128 in green. Labels provide the type and the frequency of the aa insertion; aa positions are referred to the subtype B strain BH10 (Universal Protein Knowledgebase [UniProtKB] P04585). aa, amino acid; MS, master sequences; PLWHIV, people living with human immunodeficiency virus.

Previous data obtained by analysis of MS sequences belonging to the HIV‐1 clade B, collected between 1985 and 2017 and retrieved from the Los Alamos database, indicated that the frequency of p17 HIV‐1 mutants displaying aa insertions at positions 117–118 or 125–126 has been increasing over time (Caccuri, Messali, et al. 2022). This finding attests to the circulation of virions expressing vp17 among PLWHIV, which may progressively replace those expressing refp17. To determine whether MS displaying vp17 may result from an adaptation of quasi‐species, we performed p17 sequencing by NGS in plasma samples from a cohort of viremic HIV‐1 subtype B–infected patients, collected between 1997 and 2020 (*n* = 118). The analysis showed that viruses belonging to PLWHIV in the > 2010 cohort showed a higher percentage (50.9%) of vp17s as compared with those detected in the < 2010 cohort (33.8%) (Figure 2A, red bars). Subsequently, we repeated the analysis by grouping sequences according to the p17 detected at the MS level: refp17 MS and vp17 MS. As shown in Figure 2A, the frequency of quasi‐species expressing vp17 increased over time in both groups (blue and green bars represent refp17 MS and vp17 MS, respectively). It is worth noting that expression of vp17 quasi‐species was not related to HIV‐1 viral load (Figure S1A), being it similar in both cohorts, even among experienced and naïve patients (Figures S1B,C, respectively). This analysis confirms that the circulation of vp17‐expressing virions among PLWHIV is increasing and, at the same time, indicates a rise in HIV‐1 quasi‐species expressing vp17.

The distribution and the type of vp17s aa insertions, at quasi‐species level, detected in the plasma samples collected from the < 2010 cohort and from the > 2010 cohort are shown in Figure 2B,C, respectively. A clearly distinct pattern of aa insertions was observed in the two cohorts. Indeed, sequences belonging to < 2010 group displayed three distinct point of insertion falling at position 117–118, 122–123, and 125–126, with a frequency of 52.3% of total aa insertions found at position 117–118 (Figure 2B). The > 2010 group, shown in Figure 2C, showed a less complex and heterogeneous pattern of vp17s with multiple aa inserted at different positions within the C‐terminal region (64.3%, 32.1%, and 3.6% at positions 117–118, 125–126, and 127–128, respectively).

### 3.3 PLWHIV Possess a Higher Number of vp17 Quasi‐Species When Display a vp17 MS

**Figure 3:**
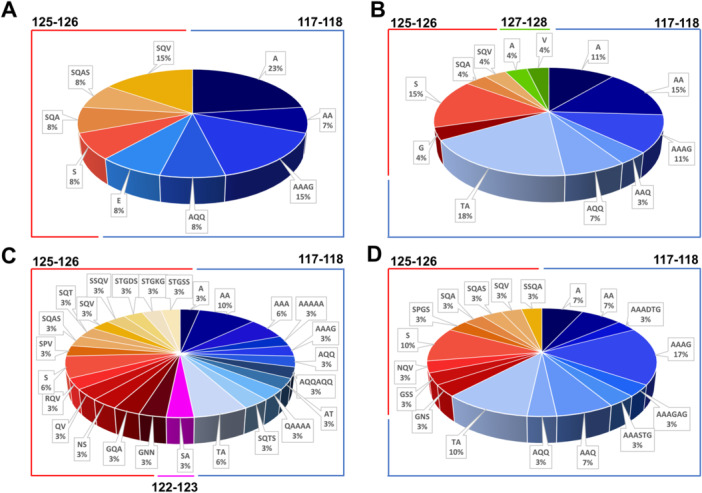
Percentage distribution of aa insertion along p17 sequences of PLWHIV. Pie charts representing the frequency of aa insertion in PLWHIV displaying refp17 MS harboring quasi‐species belonging to (A) < 2010 cohort and (B) the > 2010 group. Pie charts representing the frequency of aa insertion in PLWHIV displaying vp17s MS harboring quasi‐species within the (C) < 2010 cohort and (D) to the > 2010 group. Each insertion position is identified by a color code: position 117–118 in blue, 122–123 in pink, 125–126 in red, and 127–128 in green. Labels specify the type and the frequency of the aa insertion; aa positions are referred to the subtype B strain BH10 (Universal Protein Knowledgebase [UniProtKB] P04585). aa, amino acid; MS, master sequences; PLWHIV, people living with human immunodeficiency virus.

The distribution and type of p17 aa insertions detected in the plasma of PLWHIV harboring refp17 MS in the < 2010 cohort (Figure 3A) and the > 2010 group (Figure 3B) or in patients endowing vp17 MS in the < 2010 cohort (Figure 3C) and the > 2010 group (Figure 3D) are shown in Figure 3. The analysis revealed that, within samples harboring refp17 MS, the pattern of aa insertion was better grouped in the < 2010 cohort than in the > 2010 group (Figure 3A,B). Patients belonging to the < 2010 cohort showed a lower variability among vp17 quasi‐species, as compared with the > 2010 group, exhibiting aa insertions exclusively at positions 117–118 and 125–126 (Figure 3A,B). However, in this group a clear distinct pattern of aa insertions was observed in the < 2010 cohort, in which 61.5% of total aa insertions fell at positions 117–118, while the type of aa insertion at positions 125 to 126 was more heterogeneous. The > 2010 group showed a complex and heterogeneous pattern of vp17 quasi‐species with multiple aa inserted at different positions within the C‐terminal region (67.0%, 26.0%, and 7.0% at positions 117–118, 125–126, and 127–128, respectively). By analyzing aa insertions in samples obtained from PLWHIV displaying vp17 MS, we observed an almost similar picture between samples belonging to the < 2010 cohort (Figure 3C) and to the > 2010 group (Figure 3D). Indeed, in these groups, the distribution of aa insertions along vp17 quasi‐species almost overlapped, with a slightly less complexity in the > 2010 cohort. In particular, both cohorts displayed insertions at position 117–118 (48.4% and 62.0% in the < 2010 and > 2010 cohort, respectively) and 125–126 (48.4% and 38.0% in the < 2010 and > 2010 cohort, respectively).

### 3.4 Mutational Patterns in vp17 Quasi‐Species

**Figure 4:**
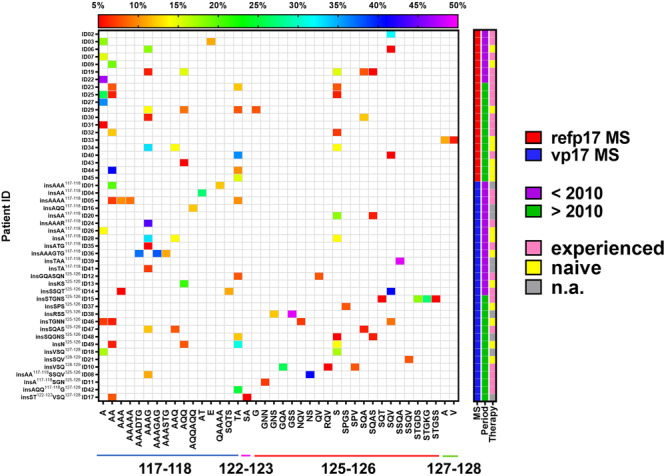
Representation of each aa insertion in p17 quasi‐species in relation to the examined characteristics of PLWHIV under evaluation. Heatmap representing vp17 quasi‐species aa insertions in each PLWHIV under evaluation. On the lower side of the heatmap from left to right the aa insertions detected in p17 quasi‐species are listed per each aa position with color code: 117–118 in blue, 122–123 in pink, 125–126 in red, and 127–128 in green. On the left side of the graph from top to bottom the analyzed PLWHIV are listed and, for the ones carrying vp17s, the insertion and its position are reported. According to the scale on the top, the intensity of the color is related to the percentage of NGS reads defining each aa insertion in the p17 quasi‐species. On the right side, the legend defines the examined characteristics of each PLWHIV, such as refp17 MS or carrying insertion, sample time of collection, and therapy administration. aa, amino acid; MS, master sequences; n.a., not available; PLWHIV, people living with human immunodeficiency virus.

To gain deeper insights into the frequency and diversity of vp17 quasi‐species detected in PLWHIV, we generated a heat map illustrating the insertions identified in each sample at positions 117–118, 122–123, 125–126, and 127–128, based on the p17 sequences of the MS across the two cohorts. Globally, 39 out of 49 (79.59%) patients harbored vp17s characterized by aa insertions at position 117–118, whereas 27 out of 49 (55.10%) exhibited insertions at position 125–126, and 1 out of 49 (2.04%) showed insertions at position 127–128 in their plasma (Figure 4). As shown in Figure 4, the majority of the insertions were detected in patient carrying a vp17 MS. Specifically, among this group, 12 out of 29 (41.37%) patients presented vp17s containing at least one aa insertion, 7 out of 29 (21.13%) displayed vp17s characterized by two aa insertions, 6 out of 29 (20.69%) exhibited vp17s endowing three aa insertions, and 4 out of 29 (13.79%) showed vp17s characterized by four aa insertions. Among PLWHIV carrying a refp17 MS, 8 out of 20 (40.00%) patients harbored vp17s characterized by at least one aa insertion, 7 out 20 (35.00%) exhibited vp17s characterized by two aa insertions, 3 out of 20 (15.00%) patients displayed vp17s carrying three aa insertions, and 2 out of 29 (6.89%) showed vp17s characterized by either four or five aa insertions. When patients were stratified according to sampling period (< 2010 and > 2010 cohort), we observed that in the < 2010 cohort, 17 out of 22 (77.27%) patients carried vp17s characterized by either one or two aa insertions, 3 out of 22 (13.63%) patients exhibited vp17s characterized by either three or five aa insertions, and 2 out of 22 (9.09%) patients displayed vp17s characterized by four aa insertions. Among PLWHIV belonging to the > 2010 cohort, 11 out of 27 (40.74%) patients harbored vp17s characterized by a single aa insertion, 6 out of 27 (22.22%) patients exhibited vp17s characterized by two aa insertions, 7 out of 27 (25.92%) patients displayed vp17s characterized by three aa insertions, and 3 out of 27 (11.11%) patients showed vp17s characterized by four aa insertions. Notably, intrasample genetic diversity, in terms of coefficient of variation, was higher in the > 2010 cohort compared with the < 2010 one [median (range), 33.16% (0.00–105.90) vs. 30.32% (0.00–100.70)], and higher among experienced patients [32.08 (0.00–100.70)] rather than among naïve subjects [30.83 (0.00–84.14)].

Consistent with previous findings (Dolcetti et al. 2015; Caccuri, Messali, et al. 2022), these results indicate that vp17s are predominantly characterized by aa insertions occurring at well‐defined positions within the C‐terminal region of the viral protein, with insertions consisting of different Alanine stretches between position 117–118 being the most frequently represented.

### 3.5 Prediction of vp17 Evolution to Dominance in the Human Host

**Figure 5:**
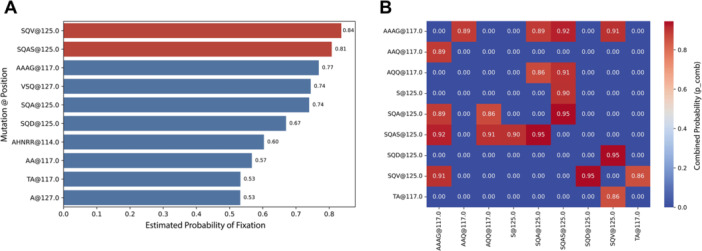
Prediction of vp17 quasi‐species evolution in the human host. (A) Top 10 refp17 MS quasi‐species with the highest predicted fixation probability. Bar plot showing the 10 refp17 MS quasi‐species with the greatest predicted likelihood of becoming fixed, based on the logistic regression model. Quasi‐species characterized by insSQV^125–126^, insSQAS^125–126^, and insAAAG^117–118^ show the highest fixation probabilities (> 0.70), indicating that the 117–127 region of the p17 protein may represent an evolutionary hotspot favoring insertion events. (B) Top 10 pairs of refp17 MS quasi‐species with the highest combined fixation probability. Heatmap showing the combined fixation probability (p_comb) for the top 10 pairs of refp17 quasi‐species. The strongest associations (> 0.90), particularly between insAAAG^117–118^ and insSQAS^125–126^, indicate potential coselection or structural compatibility among insertion‐prone variants. MS, master sequence.

Aiming at identifying insertions with the greatest potential for long‐term fixation, we focused our analysis on refp17 MS subjects to determine which genetic p17 quasi‐species are most likely to emerge and become stably inserted within the viral population over time. To this end, data derived from sequences containing insertions were used to construct a predictive model based on logistic. The model was designed to identify evolutionary patterns linked to fixed insertions and then applied to refp17 MS to estimate the probability of future fixed insertions for each quasi‐species. The analysis revealed that refp17 MS contained a limited number of quasi‐species with a high probability of becoming future insertions, predominantly located between positions 117 and 127 of the p17 protein. Notably, insertions such as insSQV^125–126^, insSQAS^125–126^, and insAAAG^117–118^ exhibited the highest fixation probabilities (> 0.70), suggesting that this region may represent an evolutionary hotspot favorable to insertion events (Figure 5A). When insertion pairs were evaluated for combined fixation risk, several combinations reached probabilities exceeding 0.90, including insAAAG^117–118^ + insSQAS^125–126^ and insSQV^125–126^ + insSQAS^125–126^, indicating potential coselection phenomena or structural compatibility between variants (Figure 5B). Overall, the model highlights a trend consistent with the increasing diversity of quasi‐species observed in more recent cohorts, reinforcing the evolutionary relevance of the 117–127 region in the adaptation of p17 to the human host.

## 4 Discussion

HIV‐1 has coevolved with its hosts, and, throughout selective pressure, viral proteins have gained and improved the specific biological functions essential for their fitness in specific hosts (Caccuri et al. 2021). Indeed, HIV‐1 mutational sites evolve under strong host's immune system selection and by viral adaptation to the new environment established by the cART (Arenas 2015). On the other hand, the rapid evolution of HIV‐1, as for most retroviruses, relies to the combined activity of error‐prone reverse transcriptase, and recombination events leading to extensive viral diversity both within and between human hosts (Ricchetti and Buc 1990; Jetzt et al. 2000). This perpetual generation of genetic variants, along with selection pressures and bottleneck events, contribute to the viral quasi‐species population generation and to the evolutionary trajectories of viruses.

In this context, HIV‐1 viral proteins may undergo biological gain‐of‐functions. It is worth noting that the HIV‐1 matrix protein p17 acquires specific aa insertions in its C‐terminal region which induce protein destabilization, leading to conformational changes that strongly impact its clonogenic B‐cell growth properties (Dolcetti et al. 2015; Caccuri, Messali, et al. 2022). This gain‐of‐function may contribute to B‐cell lymphomagenesis as suggested by the significantly higher frequency of detection of such vp17s in plasma of HIV‐1‐infected patients affected by lymphoma (Dolcetti et al. 2015; Caccuri, Messali, et al. 2022). Interestingly, aa insertions can be fixed and HIV‐1 mutant viruses displaying B‐cell clonogenic vp17s MS are actively spreading (Caccuri, Messali, et al. 2022).

Previous data obtained by analysis of HIV‐1 MS sequences indicated that the frequency of p17 HIV‐1 mutants displaying aa insertions at positions 117–118 or 125–126 has been increasing over time (Caccuri, Messali, et al. 2022). Here, through the development of a new NGS approach, we found that frequency of vp17 quasi‐species in PLWHIV increases over time leading to the generation of new MS. Indeed, analysis of p17 quasi‐species in the plasma samples from a cohort of viremic HIV‐1 subtype B–infected patients, collected between 1997 and 2020, displaying the same virological features, revealed a temporal shift in the distribution of vp17s. When comparing samples collected before < 2010 with those obtained after > 2010, the latter showed a marked higher prevalence of vp17s (50.9% vs. 33.8%). Interestingly, stratification of samples according to the p17 expressed at the MS level further demonstrated that the frequency of quasi‐species encoding vp17s increased over time in both refp17 MS and vp17 MS groups. These data support the hypothesis that the intrahost presence of vp17 quasi‐species may have driven the emergence of circulating vp17‐expressing virions. A detailed analysis showed that position 117–118 consistently represents the predominant hot spot of insertion. In the < 2010 cohort, insertions were observed at three distinct sites (117–118, 122–123, and 125–126), with 52.3% of total aa insertions occurring at position 117–118. In contrast, the > 2010 group, exhibited a less heterogeneous but still complex pattern of multiple aa inserted at different positions within the p17 C‐terminal region, with a higher frequency of insertion occurring at position 117–118 (64.3%). Within samples expressing refp17 MS, quasi‐species variability was lower in the < 2010, with insertions confined to positions 117–118 and 125–126; among these, 117–118 accounted for the majority (61.5%). The > 2010 group showed increased heterogeneous again dominated by insertions at 117–118 (67.0%). A similar distribution was observed in samples displaying vp17 MS, in which both cohorts displayed insertions almost exclusively at position 117–118 (48.4% and 62.0% in the < 2010 and > 2010 cohort, respectively) and 125–126 (48.4% and 38.0% in the < 2010 and > 2010 cohort, respectively). Collectively, these findings indicate a progressive increase of quasi‐species expressing vp17s over time and identify the aa insertion at position 117–118 as the most recurrent within the p17 C‐terminal region.

Interestingly, with the aim of identifying what are the insertions with higher potential to became stable in p17 over time, we developed a logistic regression model able to detect evolutionary patterns associated with fixed insertions. On the whole, our model identified the 117–127 region of p17 as an evolutionary hotspot. Taken together, these data support the hypothesis that the intrahost presence of fixed insertions in p17 quasi‐species—through mechanisms of host adaptation or by acquiring a greater fitness—may have led to the emergence of HIV‐1 virions expressing vp17s at the MS level.

The increased incidence of mutant viruses over time may rely to recombination events and transmission of quasi‐species. This likely stems from a recently established larger bottleneck that allows the transmission of mutants as quasi‐species that, depending on the host's characteristics, adapt to it and replicate.

HIV‐1 evolution has been driven by its matrix protein p17. Indeed, an adaptive change in this protein, at aa position 30, has been proven to be a major determinant of HIV‐1 human adaptation (Wain et al. 2007; Bibollet‐Ruche et al. 2012). Moreover, an epitope expressed on p17, spanning from aa 37–52, was recently recognized to mediate p17 angiogenic activity by interacting with β‐common chain receptor (βCR). This epitope is also shared with the matrix protein of Simian Immunodeficiency Virus originated in chimpanzees (SIVcpz) and gorillas (SIVgor), but not with that of HIV‐2 and its ancestor SIV originated in sooty mangabeys (SIVsmm). Evolution trajectory, based on this functional epitope, showed a clear differentiation between HIV‐1/SIVcpz‐gor and HIV‐2/SIVsmm branches (Caccuri et al. 2021). Therefore, acquisition of an epitope on the matrix proteins of HIV‐1 ancestors capable of triggering βCR may have represented a critical step to enhance viral aggressiveness and early human‐to‐human SIVcpz‐gor dissemination, further strengthening the key role of p17 in the evolutionary trajectory of HIV‐1 and its successful adaptation to the human host. Consequently, a defined set of structural and functional constraints maintains p17 ability to establish adaptive interactions with host factors. These constraints may act as an evolutionary mechanism enabling the virus to optimize its fitness, particularly during phases in which successful cART and immune responses impose substantial selective pressure on viral replication.

Taken together, these observations support the concept that p17 represents a key HIV‐1 evolutionary hot spot even in light of the crucial role that it plays in the HIV‐1 life‐cycle, as in proteolytic processing of Gag precursors, in early postentry steps—including nuclear import and integration—and in the assembly and egress of mature virions (Casella et al. 1997; Bukrinskaya 2007; Freed 1998).

The evolutionary trajectory of p17 leads to substantial biological consequences, as the gain of pathogenic functions. Specifically, engagement of the βCR has been implicated in conferring pro‐angiogenic properties to p17, whereas interaction with the PAR‐1 receptor has been associated with the emerging of proliferating and clonogenic vp17s that may, in turn, contribute to the pathogenesis of B‐cell lymphomagenesis in PLWHIV (Giagulli et al. 2011, Giagulli et al. 2021; Caccuri et al. 2014).

The gain‐of‐function evolution observed in vp17s is unlikely to represent a stochastic occurrence; rather, it appears to reflect a biologically directed adaptive process. The intrinsically disordered region within p17, characterized by its elevated mutational permissiveness, may confer substantial evolutionary flexibility, an attribute of particular relevance in light of the protein's diverse functional contributions to both the viral replicative framework and the modulation of drug‐resistance pathways. In this regard, a combination of deletions and mutations within the p17 protein, in proximity to the protease cleavage site, has been shown to confer significant resistance to protease inhibitors in the absence of major mutations in the viral protease (Datir et al. 2020). In conclusion, it would be of considerable interest to determine whether naturally occurring insertions in p17 may substantially modulate HIV‐1 replication dynamics and infectivity, as such insights could be very informative to develop targeted antiviral strategies and enhance our understanding of viral evolution, with potential implications for the prevention of HIV‐1‐associated lymphomagenesis. Moreover, the regression model we established can be useful as a diagnostic tool for early identification of PLWHIV at increased risk of developing lymphoma.

## Author Contributions

**Serena Messali:** writing – original draft (lead), investigation (equal), data curation (lead), formal analysis (equal), methodology (lead). **Anna Bertelli:** investigation (equal), formal analysis (equal). **Marta Giovanetti:** investigation (equal), formal analysis (equal). **Leonardo Sclavi:** investigation (equal). **Massimo Ciccozzi:** supervision (supporting). **Mark Slevin:** writing – review and editing (supporting). **Arnaldo Caruso:** conceptualization (equal), writing – review and editing (lead), supervision (supporting). **Francesca Caccuri:** conceptualization (equal), writing – review and editing (supporting), supervision (lead), funding acquisition (lead).

## Ethics Statement

The study was conducted in accordance with the Declaration of Helsinki and National Standards, and was approved by the Brescia Ethics Committee (NP 3163).

## Conflicts of Interest

The authors declare no conflicts of interest.

## Supporting information


**Figure S1:** Correlation of HIV‐1 viral load and cART therapy in PLWHIV carrying quasi‐species.

## Data Availability

All of the sequences generated in this study are available upon request.
