# Supplementary material for: Decoding HIV‐1 Next Move Through Matrix Protein p17 Quasi‐Species
Source: Microbiologyopen. 2026 Apr 3;15(2):e70280. doi: 10.1002/mbo3.70280 (PMC13052309; doi:10.1002/mbo3.70280)
Supplement: Supplementary file 1 — Figure S1: Correlation of HIV‐1 viral load and cART therapy in PLWHIV carrying quasi‐species. [file MBO3-15-e70280-s001.docx]

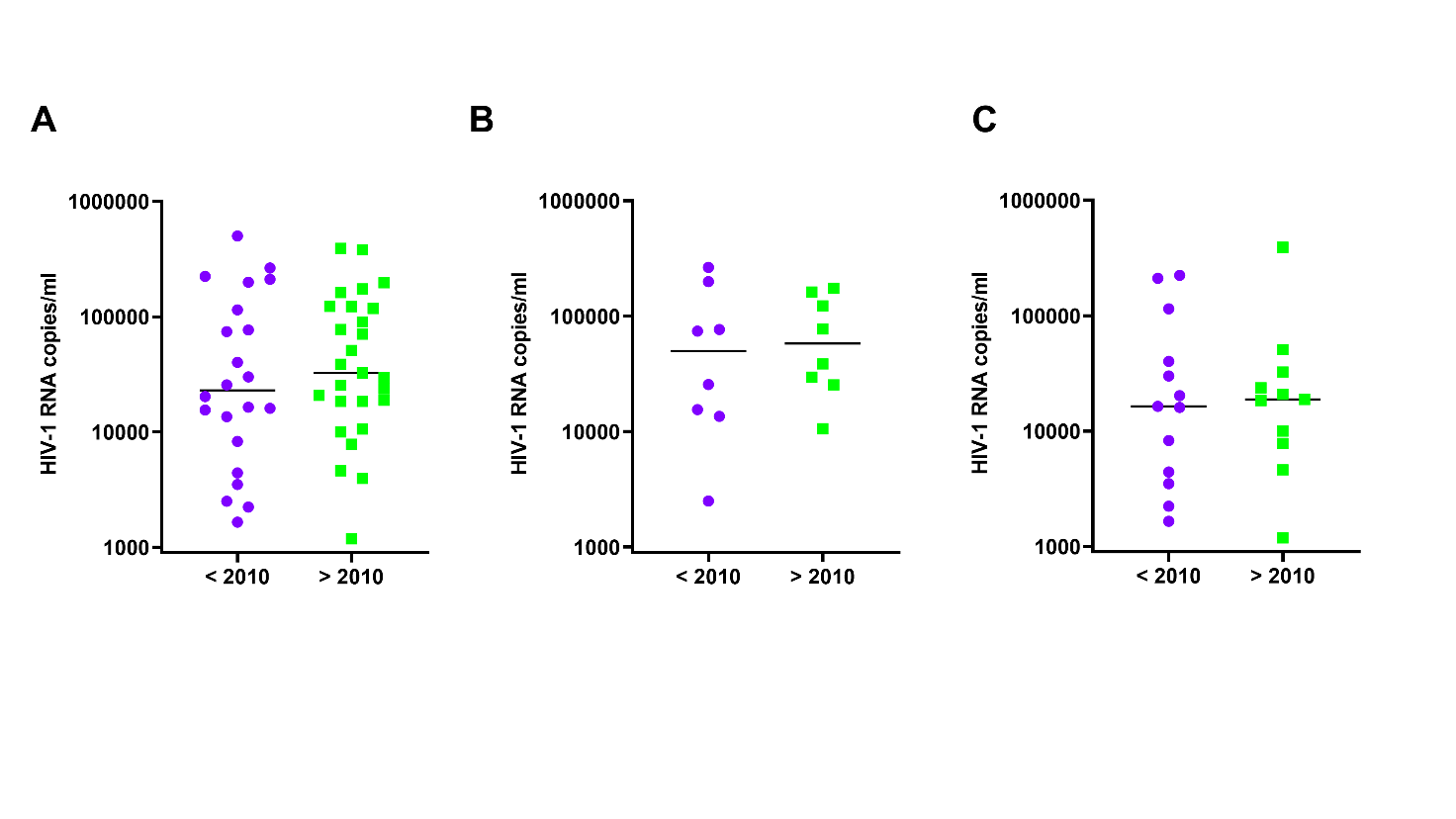


**Figure S1. Correlation of HIV-1 viral load and cART therapy in PLWHIV carrying quasi-species.** The viral load in the <2010 cohort (purple dots) and in the >2010 one (green squares) is represented in relation to the administration of cART therapy. HIV-1 viral loads levels, expressed as HIV-1 RNA copies/ml, (**A**) in the totality of the enrolled PLWHIV, (**B**) in the naïve PLWHIV and (**C**) in the experienced PLWHIV patients. cART = combined antiretroviral therapy, PLWHIV = people living with human immunodeficiency virus.
